# Urinary Phthalate Metabolites in Relation to Preterm Birth in Mexico City

**DOI:** 10.1289/ehp.0800522

**Published:** 2009-06-16

**Authors:** John D. Meeker, Howard Hu, David E. Cantonwine, Hector Lamadrid-Figueroa, Antonia M. Calafat, Adrienne S. Ettinger, Mauricio Hernandez-Avila, Rita Loch-Caruso, Martha María Téllez-Rojo

**Affiliations:** 1 Department of Environmental Health Sciences, University of Michigan School of Public Health, Ann Arbor, Michigan, USA; 2 Division of Statistics, Center for Evaluation Research and Surveys, National Institute of Public Health, Cuernavaca, Mexico; 3 Centers for Disease and Control and Prevention, Atlanta, Georgia, USA; 4 Department of Environmental Health, Harvard School of Public Health, Boston, Massachusetts, USA; 5 Ministry of Health, Mexico City, Mexico

**Keywords:** case–control, environment, epidemiology, exposure, pregnancy, prematurity

## Abstract

**Background:**

Rates of preterm birth have been rising over the past several decades. Factors contributing to this trend remain largely unclear, and exposure to environmental contaminants may play a role.

**Objective:**

We investigated the relationship between phthalate exposure and preterm birth.

**Methods:**

Within a large Mexican birth cohort study, we compared third-trimester urinary phthalate metabolite concentrations in 30 women who delivered preterm (< 37 weeks of gestation) with those of 30 controls (≥ 37 weeks of gestation).

**Results:**

Concentrations of most of the metabolites were similar to those reported among U.S. females, although in the present study mono-*n*-butyl phthalate (MBP) concentrations were higher and monobenzyl phthalate (MBzP) concentrations lower. In a crude comparison before correcting for urinary dilution, geometric mean urinary concentrations were higher for the phthalate metabolites MBP, MBzP, mono(3-carboxylpropyl) phthalate, and four metabolites of di(2-ethyl-hexyl) phthalate among women who subsequently delivered preterm. These differences remained, but were somewhat lessened, after correction by specific gravity or creatinine. In multivariate logistic regression analysis adjusted for potential confounders, elevated odds of having phthalate metabolite concentrations above the median level were found.

**Conclusions:**

We found that phthalate exposure is prevalent among this group of pregnant women in Mexico and that some phthalates may be associated with preterm birth.

Approximately 4 million neonatal deaths occur each year globally, of which more than one-quarter are caused by preterm birth (Lawn et al. 2005). Although 99% of neonatal deaths arise in low- or medium-income countries, preterm birth has also emerged as a public health priority in the United States and other industrialized nations. The U.S. rates of preterm birth have increased by > 30% since 1981 and by 18% since 1990, and as of 2004 accounted for 12.8% of live births (Martin et al. 2008). Preterm birth is associated with more than one-third of infant deaths in the United States, making it the leading cause of neonatal mortality (Mathews et al. 2004). In addition, many complications from preterm birth develop into chronic health conditions such as blindness, deafness, cerebral palsy, and lower IQ (Slattery and Morrison 2002).

There is growing concern for the potential role of environmental pollutants in preterm birth, yet this area remains poorly understood (Institute of Medicine 2006). Only a few environmental agents have been investigated for associations with preterm birth in well-controlled epidemiologic studies, including the insecticide DDT (dichlorodiphenyltrichloroethane) (Longnecker et al. 2001), secondhand tobacco smoke (Jaakkola et al. 2001; Kharrazi et al. 2004), and lead (Andrews et al. 1994). There is further evidence for the contribution of environmental exposures on preterm birth through observations of increased risk among individuals with polymorphisms in genes involved in xenobiotic metabolism (Nesin 2007).

Phthalates are a group of chemicals used in a wide range of industrial applications and consumer products, and this widespread use results in exposure among most of the general population (Hauser and Calafat 2005; Silva et al. 2004). Sources of exposure to low-molecular-weight phthalates [e.g., diethyl phthalate and di-*n*-butyl phthalate (DBP)] may include personal care products such as perfumes, lotions, and cosmetics; through their use as solvents; or as coatings on timed-release pharmaceuticals (Hauser and Calafat 2005). Exposure to higher-molecular-weight phthalates such as di(2-ethylhexyl) phthalate (DEHP) and butylbenzyl phthalate (BBzP) may be related to their use as plasticizers in flexible vinyl plastic consumer products (e.g., construction materials, floor covering, and vinyl wall paper), food packaging, and medical devices (Hauser and Calafat 2005). Exposure to DEHP was associated with decreased gestational age in studies conducted in Italy (Latini et al. 2003) and in New York City (Whyatt et al. 2008). Conversely, a different study conducted in New York City reported a positive association between gestational age and urinary concentrations of metabolites of DEHP as well as the phthalate metabolites mono-*n*-butyl phthalate (MBP) and monoethyl phthalate (MEP) (Wolff et al. 2008).

Although previous studies investigated the association between phthalate exposure and gestational age among primarily term births, the relationship with preterm births has not yet been addressed. In the present study we conducted a nested case–control analysis of the association between urinary phthalate metabolites and preterm birth in a Mexico City birth cohort.

## Materials and Methods

### Study population

The present study was nested within a Mexican birth cohort study in which women were recruited during prenatal visits at one of four clinics of the Mexican Institute of Social Security (IMSS) in Mexico City between 2001 and 2003 (Ettinger et al. 2009). The clinics serve a low- to moderate-income population. Women were eligible if they had a confirmed pregnancy of no more than 14 weeks’ gestation, did not present with a high-risk pregnancy (including daily consumption of alcoholic beverages; addiction to illegal drugs; continuous use of prescription drugs; or diagnosis of multiple pregnancy, preeclampsia, renal or heart disease, gestational diabetes, or seizures that require medical treatment), planned to reside in Mexico City for approximately 5 years, and were willing to participate in a follow-up study protocol. Gestational length was estimated by maternal recall of the date of last menstrual period, because early ultrasound is not routinely performed at the IMSS clinics. Information on demographic, socioeconomic, and other factors that could confound the relationship between phthalate exposure and gestational length was collected through questionnaire. The study was described in detail to all participating mothers, and all study participants gave informed consent. The research protocol was approved by the Ethics and Research Committees of all participating institutions. The involvement of the Centers for Disease Control and Prevention (CDC) laboratory was limited and was determined not to constitute engagement in human subjects research. Of the 1,853 women approached, 670 (36%) agreed to participate in the cohort study. Of these, archived third-trimester urine samples were available for 518 nonsmoking women because urine collection did not commence until after the initiation of study recruitment had begun. In the present nested case–control study conducted within these 518 women, 30 cases were selected randomly among 44 women who delivered before the completion of 37 weeks of gestation. Controls (*n* = 30) were selected randomly among women who had completed ≥ 37 weeks of gestation at the time of delivery.

### Phthalate metabolites in urine

A spot (second morning void) urine sample was collected from each woman during a third-trimester visit to the project’s research center. Phthalate metabolites were measured in urine to avoid potential sample contamination from the parent diester and because the metabolites, as opposed to the parent diesters, seem to be the active toxicants (Li et al. 1998; Peck and Albro 1982). The following 11 phthalate metabolites were measured at the CDC (Atlanta, GA, USA): mono(2-ethylhexyl) phthalate (MEHP), monobenzyl phthalate (MBzP), MBP, MEP, mono-isobutyl phthalate (MiBP), three oxidized metabolites of DEHP [mono(2-ethyl-5-hydroxyhexyl) phthalate (MEHHP), mono(2-ethyl-5-ox-ohexyl) phthalate (MEOHP), and mono(2-ethyl-5-carboxypentyl) phthalate (MECPP)], mono(3-carboxypropyl) phthalate [MCPP; an oxidized metabolite of both DBP and di-*n*-octyl phthalate (DOP)], and monocarboxyisooctyl phthalate (MCOP) and monocarboxyisononyl phthalate (MCNP) (oxidized metabolites of diisononyl phthalate and diisodecyl phthalate, respectively).

The analytical approach involved enzymatic deconjugation of the metabolites from their glucuronidated form, solid-phase extraction, separation with high-performance liquid chromatography, and detection by isotope-dilution tandem mass spectrometry (Silva et al. 2007). The limits of detection (LODs) were in the low nanogram per milliliter range for each phthalate metabolite. Isotopically labeled internal standards and conjugated internal standards were used to increase precision of measurements. Along with the unknown samples, each analytical run included calibration standards, reagent blanks, and quality control materials of high and low concentration to monitor for accuracy and precision. Analysts at the CDC were blind to all information concerning subjects.

Urinary phthalate metabolite concentrations were corrected for urine dilution by specific gravity (SG) using the formula *P*_c_ = *P*[(1.014 − 1)/*SG* − 1)], where *P*_c_ is the SG-corrected phthalate metabolite concentration (micrograms per liter), *P* is the observed phthalate metabolite concentration, 1.014 is the median SG value among the present study population, and *SG* is the specific gravity of the individual urine sample. SG was measured using a handheld digital refractometer (ATAGO Company Ltd., Tokyo, Japan). Phthalate metabolite concentrations were also corrected by creatinine (Cr; phthalate metabolite concentrations expressed as micrograms per gram Cr), which was measured using a MicroLab AT Plus robotic liquid handler (Hamilton Co., Reno, NV, USA) and Microplate Spectrophotometer (SpectraMax 340; Molecular Devices, Sunnyvale, CA, USA). Finally, we calculated (in nanomoles per milliliter) the sum of concentrations of DEHP metabolites that were measured (i.e., MEHP, MEHHP, MEOHP, and MECPP).

### Statistical analysis

We performed data analysis using SAS version 9.1 (SAS Institute Inc., Cary, NC, USA). Maternal and pregnancy characteristics were tabulated and compared between cases and controls using Student’s *t*-tests and chi-square tests when appropriate. For phthalate metabolite concentrations below the LOD, we used an imputed value equal to one-half the LOD. In preliminary crude analyses to assess whether women who experienced a preterm birth had higher phthalate metabolite concentrations than controls, we compared geometric mean values between the two groups using *t*-tests. To take into account potential confounding variables, we used multivariable logistic regression to model the odds of having high (above the median) phthalate metabolite concentrations among the preterm women compared with controls. Variables considered in this analysis included maternal age, prepregnancy body mass index (BMI), parity, education, marital status, the infant’s sex, and gestational age at the time of urine sample collection. Variables that did not differ between cases and controls and did not statistically confound (change in effect estimate of > 10%) in any of the models were not retained. The variables that did appreciably change (> 10%) the effect estimate in at least one of the models were included in all models for consistency.

## Results

Table 1 presents maternal and pregnancy characteristics of the 30 selected preterm cases and the 30 term controls. Cases and controls did not differ by age, BMI, parity, or history of previous preterm deliveries. Cases and controls differed slightly with regard to marital status, education, and gestational age at the time the urine sample was collected. Table 2 presents distributions of uncorrected phthalate concentrations measured in urine of controls. Similar to recent U.S. studies, phthalate metabolites were detected in all samples. Among monoesters, the lower-molecular-weight phthalate metabolites MBP and MEP had median values of 33.4 and 108 μg/L, respectively. In comparison, the higher-molecular-weight monoester MBzP had a median concentration of 2.85 μg/L. Among DEHP metabolites, the oxidative metabolites MEOHP, MEHHP, and MECPP had median concentrations of 13.6, 17.1, and 38.2 μg/L, respectively, which, consistent with previous reports (CDC 2005), were higher than that of the monoester MEHP (3.0 μg/L).

Correlations between phthalate metabolites were moderate to strong. Spearman correlation coefficients between metabolites from different parent diesters ranged from 0.21 (between MEHP and MEP) to 0.68 (between MECPP and MCPP). The Spearman correlation between MEHP and MBP was 0.44. Among the DEHP metabolites, Spearman correlations ranged from 0.80 to 0.84 between MEHP and the oxidized metabolites (MEHHP, MEOHP, and MECPP) and were ≥ 0.91 between the oxidized DEHP metabolites. Among metabolites of DBP, the Spearman correlation between MBP and MCPP was 0.85.

Table 3 presents crude results comparing phthalate metabolite concentrations between cases and controls for phthalate concentrations that were uncorrected for urine dilution, SG corrected, and Cr corrected. When phthalates were uncorrected for dilution, concentrations of all metabolites detected in at least 80% of samples were higher among cases compared with controls, and concentrations for many metabolites among cases were nearly double those of controls. The largest difference in uncorrected phthalate urinary metabolites was observed with MBP, for which median concentrations of cases were 3-fold higher than controls (*p* < 0.005). Cases had higher SG (*p* = 0.07) and Cr (*p* = 0.06) values than did controls, indicating that the urine samples of women who delivered at term were more diluted than samples from women who delivered preterm. These differences in urine dilution somewhat reduced the observed differences in phthalate metabolite concentrations after correcting by SG and Cr, but concentrations of all metabolites remained higher in cases than in controls.

Table 4 presents results from multivariable logistic regression analysis adjusted for potential confounding variables. Preterm birth cases had elevated odds for having phthalate metabolite concentrations above the median for all metabolites. For metabolite concentrations uncorrected for dilution, the greatest odds ratios (ORs) and 95% confidence intervals (CIs) were observed for the DBP and diisobutyl phthalate metabolites MBP (OR = 10.7; 95% CI, 2.4–47.4), MiBP (3.6; 1.1–12.2), and MCPP (6.3; 1.8–21.9); for the DEHP metabolites MEHP (3.5; 1.0–12.9), MEHHP (4.6; 1.3–16.7), and MEOHP (7.1; 1.9–26.5); and for the sum of DEHP metabolites (5.0; 1.4–18.0). Correction of metabolite concentrations by SG or Cr attenuated these ORs, although they remained elevated for most metabolites, particularly MBP and MEHP.

## Discussion

We conducted a nested case–control analysis of Mexican mother–infant pairs and found evidence for the presence of higher third-trimester urinary concentrations of phthalate metabolites among pregnant women who delivered pre-term (< 37 weeks gestation) compared with women who delivered at term (≥ 37 weeks). The differences between cases and controls for DBP metabolites (MBP and MCPP; MCPP is an oxidative metabolite of DBP as well as a major metabolite of DOP) and MEHP were not sensitive to correction for urinary dilution using SG or Cr or to the adjustment for potential confounding variables. These results provide the first evidence of an association between phthalate exposure during pregnancy and preterm birth.

Our findings of higher DEHP metabolite concentrations (MEHP and the oxidative metabolites MEOHP, MEHHP, and MECPP) among mothers delivering pre-term are consistent with an Italian study of 84 newborns that reported an inverse association between gestational age and DEHP exposure (Latini et al. 2003). In that study, MEHP (but not DEHP) in the cord blood of the newborns was associated with decreased gestational age at delivery (OR for absence of detectable MEHP in cord blood associated with a 1-week increase in gestational age = 1.50; 95% CI, 1.01–2.21), and the mean gestational age among infants with MEHP detected in cord blood was 1.2 weeks (8.4 days) less than that of infants with nondetectable MEHP in cord blood (*p* = 0.03). However, there are uncertainties over the use of cord blood to measure phthalates in the study, because blood measures of phthalate exposure can be compromised because of contamination by phthalate diesters in sampling tubes and lipases in the blood that can convert the diesters to monoesters (Kato et al. 2003).

Our finding that DEHP metabolites were elevated in mothers delivering preterm is also consistent with a recent study of 331 African-American and Dominican mothers and newborns in New York City (Whyatt et al. 2008). In that study, women with third-trimester spot urine samples with SG-corrected MEHP concentrations in the highest quartile delivered infants with gestational ages 5.1 days (95% CI, 2.1–8.4 days) less than did women in the lowest MEHP quartile (*p* < 0.001). In contrast, our results are inconsistent with another recent study of third-trimester urines from 404 women in a multiethnic birth cohort from New York City, which reported increased gestational ages in relation to MEHP and the sum of MBP, MEP, and MiBP concentrations (Wolff et al. 2008). Differences between the results of the two New York City studies and between our study and the study by Wolff et al. (2008) may reflect differences in study designs, population characteristics (e.g., age, ethnicity, education, and quality of care), exclusion criteria, and/or exposure levels or sources. Moreover, gestation length, but not preterm birth, was assessed in these previous epidemiologic studies of phthalates and birth outcomes.

There were some differences in phthalate metabolite concentrations among pregnant women who delivered at ≥ 37 weeks in our Mexican cohort compared with those reported in the 2001–2002 National Health and Nutrition Examination Survey for women in the United States (Adibi et al. 2008; CDC 2005). The median MBP concentration uncorrected for urine dilution was 2-fold higher in our Mexican cohort than in females from the U.S. population, and the Cr-corrected MBP median was 3-fold higher. Conversely, median MBzP concentrations were about 4-fold lower in the present study compared with U.S. females for uncorrected and Cr-corrected concentrations. Median uncorrected MEHP, MEHHP, MEOHP, MiBP, MCPP, and MEP concentrations were comparable between the two populations, although somewhat lower among women in the present study. The presence of similar to slightly lower MiBP concentrations but higher MBP concentrations in this Mexican population suggests potential differences in patterns or sources of exposure to dibutyl phthalates compared with U.S. women. Based on our preliminary exposure results and evidence for adverse effects of DBP and/or MBP on fetal development (Foster 2006; Swan 2008), research designed to assess sources of DBP exposure among pregnant Mexican women is warranted.

Compared with controls, women who had preterm births in the present study had suggestively higher levels of urinary Cr [and SG, because the two were highly correlated (Spearman *r* = 0.9)], which served to lessen the differences in phthalate metabolite concentrations after correcting for SG or Cr. Cr clearance increases significantly during pregnancy, in a manner that peaks and then begins to decline several weeks before term (Boeniger et al. 2003; Davison et al. 1980; Lohsiriwat and Imrittha 2008; Sims and Krantz 1958). There is variability in the magnitude and timing of the peaks (as well as the slope of the subsequent decline in Cr clearance) among women (Davison et al. 1980; Sims and Krantz 1958), and it may not be appropriate to correct third-trimester urinary phthalate metabolite concentrations by Cr (Adibi et al. 2008). It is possible that the higher Cr levels among cases may be related to an unmeasured risk factor for preterm birth, but to our knowledge the ability of urinary Cr to predict pre-term birth has not been previously reported. In addition to fluctuations in Cr clearance during pregnancy, there are also cyclic within-woman fluctuations in Cr clearance in relation to normal menstrual cycles that are potentially caused by changes in hormone activity (Davison and Nobles 1981). Thus, it may also be possible that increased urinary Cr levels could be related to phthalate-induced alterations in endocrine function, although there are currently no data to support this hypothesis.

Preterm birth is likely a syndrome with multiple etiologies (Goldenberg et al. 2008). Although there is no clear explanation at this time for an association between phthalate exposure and preterm birth, several plausible hypotheses may be put forth. Among the various risk factors identified for preterm birth, maternal inflammation, particularly chorioamnionitis, has been firmly linked to preterm birth in epidemiologic and laboratory studies (Romero et al. 2007). Because phthalate metabolites stimulate proinflammatory responses in cells in culture, including cytokine release (Jepsen et al. 2004), activation of the mitogen-activated protein kinase pathway (Pauley et al. 2002), and activation of the peroxisome proliferator-activated receptor (PPAR)-α and PPAR-γ pathways (in a rat placental trophoblast cell line) (Xu et al. 2005), phthalate exposure may increase risk for preterm birth if it stimulates an inflammatory response in pregnant women, as proposed previously (Latini et al. 2005, 2006). Additionally, prostaglandins are important signaling molecules in parturition, and phthalate metabolites increase expression of prostaglandin-endoperoxide synthase 2 (PTGS2, also known as COX-2) in a rat placental trophoblast cell line (Xu et al. 2005) and mouse liver cells (Ledwith et al. 1997). Because PTGS2 is necessary for synthesis of prostaglandins, phthalate-stimulated induction of PTGS2 in gestational tissues such as placenta and the extraplacental membranes could increase risk for preterm birth by increasing premature intrauterine production of prostaglandins. Alternatively, matrix metalloproteinases (MMPs) are activated by inflammatory cytokines, and MEHP activates an MMP in rat testis (Yao et al. 2009). If phthalate metabolites activate MMPs in extraplacental membranes, it could lead to preterm premature rupture of the membranes (PPROM) and preterm birth.

In addition to inflammation, increased apoptosis in the maternal/fetal membranes is associated with PPROM as well as membrane rupture at term (Kataoka et al. 2002; Reti et al. 2007). MEHP stimulates cell responses that can initiate apoptosis, including DNA damage (in human sperm) (Hauser et al. 2007), oxidative stress (in the testes) (Kasahara et al. 2002), release of cytochrome c from mitochondria (Kasahara et al. 2002), and increased expression of Fas ligand, an apoptosis-initiating protein (Richburg and Boekelheide 1996; Yao et al. 2007). Oxidative stress is also believed to be involved in the developmental toxicity of DBP (Kim et al. 2002; Wellejus et al. 2002). However, most studies of phthalate metabolite–stimulated oxidative stress, DNA damage, and apoptosis have been conducted in males, and studies of effects in females and on pregnancy outcomes are needed.

Alternate mechanisms could involve phthalate impacts on endocrine function. The phthalate diesters DEHP, DBP, and BBzP, and/or their metabolites, are antiandrogenic in males (Foster 2006; Meeker et al. 2008; Swan 2008) and have been shown to reduce estradiol and progesterone production in female rodents and in rat granulosa cells *in vitro* (Gray et al. 2006; Lovekamp and Davis 2003; Treinen et al. 1990). Because progesterone plays a key role in maintaining uterine quiescence during pregnancy, it may be plausible that phthalates alter the timing of labor by reducing progesterone production. Furthermore, estradiol is vital in suppressing inflammation and oxidative stress (Straub 2007; Vina et al. 2006). However, data in support of the pathways proposed here involving endocrine disturbances, inflammation, and oxidative stress are lacking, and additional research is needed to explore these potential mechanisms.

The present study has several limitations, including its relatively small size, the inability to study high-risk preterm births occurring more remote from term, and the potential for misclassification stemming from maternal recall of last menstrual period to estimate gestational age. Misclassification of preterm cases may be nondifferential with respect to exposure status. However, we cannot rule out differential misclassification. For example, although there is currently no evidence in support of this hypothesized scenario, if phthalate exposure was associated with altered menstrual cycle length, it may have resulted in systematic underestimates of last menstrual period and gestational age among highly exposed women. Another limitation of our study is the potential for uncontrolled biases due to the 1-week difference in median gestational age at the time of urine sample collection between cases and controls. However, although this sample timing was associated with case status, and statistically confounded at least one of the multivariable models and thus was included in the adjusted ORs presented in Table 4, it was not associated with uncorrected or dilution-corrected urinary phthalate metabolite concentrations. Our study also made a large number of statistical comparisons, which may have led to chance findings of statistical significance. In addition, the potential for reverse causation cannot be ruled out, whereby other underlying risk factors for delivering preterm could be associated with increased phthalate exposure or altered phthalate metabolism. Finally, our ability to interpret these results to determine which specific phthalates or phthalate metabolites may be the most relevant to preterm birth risk was limited because concentrations of many compounds were higher among preterm cases and the various phthalate metabolites were moderately to strongly correlated with one another.

In conclusion, the present study provides evidence for a potential role for phthalate exposure (DBP, DOP, DEHP, and/or BBzP) in pre-term birth among a group of Mexican women. Additional research, including larger human studies and experimental studies, are warranted to further investigate the relationship between phthalate exposure and preterm birth.

## Figures and Tables

**Table 1:** Maternal and pregnancy characteristics for controls (term) and cases (preterm).

Characteristic	Term (≥ 37 weeks; *n* = 30)	Preterm (< 37 weeks; *n* = 30)
Maternal age (years) [median (25th, 75th percentile)]	27 (23, 30)	27 (23, 32)
Prepregnancy BMI [median (25th, 75th percentile)]	25 (22, 27)	25 (23, 28)
Weeks gestation at urine sample [median (25th, 75th percentile)]	34 (33, 35)	33 (32, 35)
Parity [no. (%)]		
First child	10 (33)	10 (33)
Not first child	20 (67)	20 (67)
Previous preterm delivery [no. (%)]		
Yes	2 (7)	2 (7)
No	28 (93)	28 (93)
Marital status [no. (%)]		
Married	20 (67)	17 (57)
Unmarried living with partner	6 (20)	7 (23)
Single	4 (13)	6 (20)
Maternal education [no. (%)]		
< High school	17 (57)	15 (50)
≥ High school	13 (43)	15 (50)
Infant sex [no. (%)]		
Male	15 (50)	13 (43)
Female	15 (50)	17 (57)

**Table 2:** Distribution of third-trimester urinary concentrations of phthalate metabolites (uncorrected for dilution) among 30 Mexican women who delivered ≥ 37 weeks of gestation (μg/L).

Phthalate metabolite	% > LOD	Geometric mean	Percentile					
			25th	50th	75th	90th	95th	Maximum
MEHP	80	1.9	0.60	3.00	4.40	9.55	11.8	14.1
MEHHP	100	13.6	6.20	17.1	28.4	65.7	91.5	129
MEOHP	100	10.4	5.00	13.6	24.5	49.6	74.2	101
MECPP	100	29.7	14.3	38.2	53.8	135	160	173
MBzP	92	2.3	1.00	2.85	5.20	16.8	20.2	23.3
MBP	100	38.1	21.3	33.4	74.0	198	355	557
MiBP	97	1.9	0.80	2.00	4.10	8.80	13.1	58.5
MCPP	95	1.1	0.50	1.25	2.00	4.10	5.10	9.80
MCOP	67	NC	< LOD	0.80	1.20	2.70	3.70	4.90
MCNP	67	NC	< LOD	0.85	1.20	1.80	2.40	2.80
MEP	100	112	47.1	108	224	740	897	4,750
Sum DEHP^a^	—	0.19	0.09	0.24	0.38	0.86	1.14	1.34

NC, not calculated because of high proportion (33%) of samples < LOD (0.7 μg/L for MCOP and 0.6 μg/L for MCNP).

a nmol/L.

**Table 3:** Geometric mean and median third-trimester urinary phthalate metabolite concentrations among women who had term or preterm births.

	Geometric mean		Median (25th, 75th percentile)		
Phthalate metabolite	Term (*n* = 30)	Preterm (*n* = 30)	Term (*n* = 30)	Preterm (*n* = 30)	*p*-Value^a^
Uncorrected (μg/L)					
MEHP	1.9	3.5	3.0 (0.3, 4.4)	4.3 (2.2, 7.1)	0.05
MEHHP	13.6	24.0	17.1 (6.2, 28.4)	28.7 (18.1, 37.5)	0.04
MEOHP	10.4	18.9	13.6 (5.0, 24.5)	20.8 (14.4, 25.5)	0.04
MECPP	29.7	51.2	38.2 (14.3, 53.8)	55.2 (39.2, 73.3)	0.02
MBzP	2.3	5.2	2.9 (1.0, 5.2)	5.4 (2.6, 9.5)	0.01
MBP	38.1	89.9	33.4 (21.3, 74.0)	97.1 (56.0, 139)	0.005
MiBP	1.9	3.1	2.0 (0.8, 4.1)	3.3 (2.2, 5.1)	0.08
MCPP	1.1	2.4	1.3 (0.5, 2.0)	2.3 (1.1, 4.9)	0.002
MCOP	NC	NC	0.80 (< LOD, 1.2)	1.2 (< LOD, 1.7)	NC
MCNP	NC	NC	0.85 (< LOD, 1.2)	0.90 (< LOD, 1.6)	NC
MEP	112	204	108 (47.1, 224)	171 (69.4, 437)	0.10
Sum DEHP metabolism	0.19	0.33	0.24 (0.09, 0.38)	0.37 (0.23, 0.45)	0.03
SG corrected (μg/L)					
MEHP	2.5	3.6	3.0 (1.1, 5.9)	4.3 (2.6, 6.2)	0.15
MEHHP	18.1	24.5	21.3 (9.3, 31.4)	25.7 (17.2, 31.4)	0.14
MEOHP	13.9	19.3	15.5 (7.8, 29.0)	20.1 (14.4, 23.8)	0.11
MECPP	39.5	52.4	37.1 (22.4, 65.2)	56.7 (38.8, 72.8)	0.09
MBzP	3.1	5.3	3.2 (1.5, 7.8)	6.5 (3.5, 9.8)	0.05
MBP	50.7	92.0	52.4 (28.4, 101)	88.1 (58.6, 148)	0.01
MiBP	2.5	3.2	2.3 (1.1, 5.0)	3.3 (1.9)	0.3
MCPP	1.4	2.4	1.6 (0.7, 2.6)	2.3 (1.4, 4.1)	0.009
MCOP	NC	NC	0.49 (< LOD, 1.3)	1.0 (< LOD, 1.5)	NC
MCNP	NC	NC	0.86 (< LOD, 1.4)	0.91 (< LOD, 1.3)	NC
MEP	150	209	134 (45.2, 284)	182 (68.3, 340)	0.3
Sum DEHP metabolism	0.25	0.34	0.27 (0.13, 0.44)	0.37 (0.24, 0.42)	0.11
Cr corrected (μg/g Cr)					
MEHP	3.3	4.7	3.7 (1.7, 7.4)	5.6 (3.3, 7.4)	0.18
MEHHP	24.1	32.1	23.0 (11.4, 52.1)	33.2 (24.1, 41.5)	0.17
MEOHP	18.5	25.3	19.0 (9.5, 42.1)	24.5 (20.6, 29.2)	0.14
MECPP	52.8	68.5	48.0 (27.3, 98.6)	71.1 (52.7, 77.4)	0.10
MBzP	4.1	7.0	4.6 (2.2, 9.1)	8.7 (4.1, 11.7)	0.05
MBP	67.8	120	63.1 (34.8, 176)	127 (81.4, 171)	0.01
MiBP	3.3	4.2	3.7 (1.3, 6.6)	4.1 (2.3, 7.9)	0.3
MCPP	1.9	3.2	1.9 (1.0, 3.2)	2.9 (1.9, 5.3)	0.007
MCOP	NC	NC	0.68 (< LOD, 1.8)	0.90 (< LOD, 1.7)	NC
MCNP	NC	NC	1.2 (< LOD, 1.7)	1.1 (< LOD, 1.5)	NC
MEP	200	274	186 (64, 401)	232 (106, 396)	0.3
Sum DEHP metabolism	0.34	0.44	0.26 (0.16, 0.55)	0.36 (0.28, 0.45)	0.13

NC, not calculated because of high proportion (33%) of samples < LOD.

a Two-tailed Student’s *t*-test of ln-transformed phthalate metabolite concentrations.

**Table 4:** Adjusted^a^ ORs for high (> median) third-trimester urinary phthalate metabolite concentrations (unadjusted, SG corrected, and Cr corrected) among women who delivered preterm compared with controls.

Phthalate metabolite	Unadjusted (μg/L)			SG corrected (μg/L)			Cr corrected (μg/g Cr)		
	*n*_1_	*n*_2_	OR (95% CI)	*n*_1_	*n*_2_	OR (95% CI)	*n*_1_	*n*_2_	OR (95% CI)
MEHP	18	12	3.5 (1.0–12.9)	18	12	3.2 (0.9–11.3)	18	12	3.2 (0.9–11.3)
MEHHP	19	11	4.6 (1.3–16.7)	15	15	0.9 (0.3–3.1)	17	13	2.9 (0.8–10.8)
MEOHP	20	10	7.1 (19–26.5)	17	13	1.9 (0.6–6.5)	17	13	3.2 (0.9–11.0)
MECPP	18	12	2.8 (0.9–9.3)	18	12	3.4 (1.0–12.0)	17	13	2.9 (0.8–11.0)
MBzP	18	12	2.5 (0.8–8.5)	18	12	2.2 (0.7–6.7)	12	12	2.2 (0.7–6.7)
MBP	20	10	10.7 (2.4–47.4)	19	11	4.5 (1.2–16.6)	20	10	5.4 (1.5–19.3)
MiBP	19	11	3.6 (1.1–12.2)	17	13	2.0 (0.7–6.0)	16	14	1.5 (0.5–4.5)
MCPP	21	9	6.3 (1.8–21.9)	19	11	3.2 (1.0–9.8)	18	12	3.0 (0.9–10.0)
MCOP	19	11	4.3 (1.2–14.9)	16	14	1.3 (0.5–3.9)	17	13	2.0 (0.7–6.0)
MCNP	18	12	1.3 (0.4–4.0)	15	15	1.2 (0.4–3.6)	15	15	1.2 (0.4–3.6)
MEP	18	12	2.3 (0.7–7.3)	16	14	1.3 (0.4–4.2)	16	14	1.3 (0.4–4.1)
Sum DEHP	19	11	5.0 (1.4–18.0)	17	13	1.9 (0.6–6.5)	17	13	4.1 (1.0–17.5)

Abbreviations: *n*_1_, number of cases with high (> median) urinary phthalate concentration; *n*_2_, number of controls with high (> median) urinary phthalate concentration.

a Adjusted for marital status, maternal education, infant sex, and gestational age at time of urine sample.
